# Initial Investigation of Transmission of COVID-19 Among Crew Members During Quarantine of a Cruise Ship — Yokohama, Japan, February 2020

**DOI:** 10.15585/mmwr.mm6911e2

**Published:** 2020-03-20

**Authors:** Kensaku Kakimoto, Hajime Kamiya, Takuya Yamagishi, Tamano Matsui, Motoi Suzuki, Takaji Wakita

**Affiliations:** ^1^Field Epidemiology Training Program, National Institute of Infectious Diseases, Tokyo, Japan; ^2^Infectious Disease Surveillance Center, National Institute of Infectious Diseases, Tokyo, Japan; ^3^National Institute of Infectious Diseases, Tokyo, Japan.

An outbreak of coronavirus disease 2019 (COVID-19) among passengers and crew on a cruise ship led to quarantine of approximately 3,700 passengers and crew that began on February 3, 2020, and lasted for nearly 4 weeks at the Port of Yokohama, Japan (*1*). By February 9, 20 cases had occurred among the ship’s crew members. By the end of quarantine, approximately 700 cases of COVID-19 had been laboratory-confirmed among passengers and crew. This report describes findings from the initial phase of the cruise ship investigation into COVID-19 cases among crew members during February 4–12, 2020.

On February 1, a laboratory-confirmed case of COVID-19 was identified in a passenger who had developed symptoms on January 23 and disembarked on January 25, before the ship arrived in Yokohama. Another passenger with a laboratory-confirmed case of COVID-19 had developed symptoms on January 22 and was on the ship when it arrived in Yokohama on February 3. All symptomatic passengers were tested upon arrival in Yokohama, and those with positive results were disembarked February 4 and 5. The index patient for this outbreak could not be determined. On February 5, passengers remaining on the ship were requested to observe 14-day quarantine in their cabins. Approximately two thirds of the persons on board were passengers staying in cabins located on decks 5–12. The remainder (N = 1,068) were crew members, >80% of whose cabins were on decks 2–4. Crew members remained on board the ship at all times and had not disembarked during port calls. After quarantine began, crew members continued to perform their regular duties, delivered meals to passengers, and remained in their cabins when they were not working; symptomatic crew members were required to remain in their cabins.

Because the first detected cases occurred among passengers who became symptomatic on January 22 and 23, COVID-19 was likely transmitted first from passengers to crew members and subsequently spread among the crew, especially among food service workers. The first case detected in a crew member occurred in a food service worker who developed fever on February 2. A real-time polymerase chain reaction test performed by the Yokohama quarantine office laboratory was positive for SARS-CoV-2, the etiologic agent of COVID-19, and the crew member was permitted to disembark in Yokohama on February 4. By February 9, a total of 20 cases* among crew members had been laboratory-confirmed, including three in those who reported close contact with other crew members with laboratory-confirmed COVID-19 before implementation of quarantine. Seven ill crew members had symptom onset within 3 days of the start of quarantine, indicating that some SARS-CoV-2 transmission likely occurred before the implementation of quarantine.

The crew dining area was identified as the primary area of congregation for the crew; passengers did not have access to this part of the ship. The earliest laboratory-confirmed COVID-19 cases in crew members occurred in food service workers; 15 of the 20 confirmed cases in crew members occurred among food service workers who prepared food for other crew members, and 16 of the 20 cases occurred among persons with cabins on deck 3, the deck on which the food service workers lived (Table). Until February 6, no mechanism for systematic testing was implemented; only crew members who visited the medical clinic with symptoms were tested, and information on the total number of tests administered is not available.

**TABLE:** Coronavirus disease 2019 (COVID-19) cases and fever status among crew members aboard a cruise ship (N = 1,068) — Yokohama, Japan, February 2020

Characteristic	No. of crew members	No. (%) febrile at the time of survey		No. (%) confirmed cases^†,§^
		Feb 3	Feb 9*	
**Type of work**				
Food service	245	0 (—)	20 (8)	15 (6)
Housekeeping	176	0 (—)	0 (—)	1 (1)^¶^
Galley	135	0 (—)	3 (2)	0 (—)
Beverage service	61	0 (—)	2 (3)	2 (3)
Deck	57	1 (2)	2 (4)	0 (—)
Steward	53	0 (—)	1 (2)	1 (2)
Guest service	40	1 (3)	1 (3)	0 (—)
Gift shop	28	1 (4)	0 (—)	0 (—)
Production cast	27	0 (—)	1 (4)	0 (—)
Arts	5	0 (—)	1 (20)	0 (—)
Others	241	0 (—)	0 (—)	1 (—)
**Total**	**1,068**	**3 (0.3)**	**31 (3)**	**20 (2)**
**Crew member cabin deck**				
2	171	0 (—)	2 (1)	2 (1)
3	582	1 (0.2)	26 (4)	16 (3)
4	148	1 (1)	1 (1)	1 (1)
5	84	0 (—)	1 (1)	1 (1)
6	33	0 (—)	1 (3)	0 (—)
7	30	1 (3)	0 (—)	0 (—)
Others	18	0 (—)	0 (—)	0 (—)
N/A	2	0 (—)	0 (—)	0 (—)
**Total**	**1,068**	**3 (0.3)**	**31 (3)**	**20 (2)**

**Abbreviation:** N/A = not available.

* The survey conducted on February 9 did not include a crew member who had disembarked.

**^†^** Food service worker crew members were more likely to be infected than were housekeeping or galley crew members (Bonferroni multiple comparison test: p<0.005).

^§^ Testing for COVID-19 was conducted for crew members who sought medical attention.

^¶^ Although this crew member did not report fever, other symptoms and close contact with another patient with confirmed COVID-19 were reported, which led to testing for COVID-19.

The cruise ship company administered a questionnaire to all crew members on February 3, at which time three crew members reported subjective fever. A second survey of crew members was conducted on February 9, at which time fever was reported by 31 crew members, 20 (65%) of whom were food service workers.

Interviews were conducted with nine crew members with confirmed COVID-19 on February 12, just before their disembarkation; three of these patients reported close contact with other crew members with confirmed COVID-19 before their symptoms began. These interviews indicated that infection had apparently spread among persons whose cabins were on the same deck (deck 3) and who worked in the same occupational group (food service), probably through contact or droplet spread, which is consistent with current understanding of COVID-19 transmission (*2*). Eight of 20 crew members with confirmed COVID-19 had cabin mates; investigators later learned that following disembarkation, as of March 4, five of the eight cabin mates had also developed COVID-19.

This investigation underscores the need for swift epidemiologic investigation as soon as a COVID-19 case is detected in an area or group where a large number of persons gather in a closed or crowded setting (e.g., a cruise ship, music club, health care setting, sports arena, or gymnasium). These settings have been previously associated with infections spread by contact or droplet, such as influenza (*3*). Close contacts of persons with confirmed COVID-19 should self-quarantine and monitor their symptoms; persons who develop COVID-19 symptoms while on board a ship should be isolated to limit transmission to other passengers and crew.^†^

## Footnotes

* Testing for COVID-19 was conducted for crew members who sought medical attention.

† https://www.cdc.gov/quarantine/maritime/recommendations-for-ships.html.
