# *Erratum:* Vol. 69, No. 11

**DOI:** 10.15585/mmwr.mm6913a5

**Published:** 2020-04-03

In the report “Initial Investigation of Transmission of COVID-19 Among Crew Members During Quarantine of a Cruise Ship — Yokohama, Japan, February 2020,” on page 312, the second sentence of the first complete paragraph in the second column should have read “The earliest laboratory-confirmed COVID-19 cases in crew members occurred in food service workers; 15 of the 20 confirmed cases in crew members occurred among food service workers who prepared food for other crew members **and passengers**, and 16 of the 20 cases occurred among persons with cabins on deck 3, the deck on which the food service workers lived (Table).”

